# Supplementary material for: Choroid Plexus Modulates Subventricular Zone Adult Neurogenesis and Olfaction Through Secretion of Small Extracellular Vesicles
Source: Res Sq. 2025 Dec 5:rs.3.rs-8176257. Preprint. [Version 1] doi: 10.21203/rs.3.rs-8176257/v1 (PMC12687813; doi:10.21203/rs.3.rs-8176257/v1)
Supplement: Supplement 1 [file NIHPPRS8176257V1-supplement-1.pdf]

Table S1. Summary of Resources for Key Chemicals, Reagents and Assays Used in this Report.

REAGENT or RESOURCE	SOURCE	IDENTIFIER
**Antibodies**		
Rabbit anti-MAP2 polyclonal	Proteintech	17490-1-AP
Chicken anti-Nestin polyclonal	Novus	NB100-1604
Rabbit anti-GFAP	Abcam	ab7260
Rabbit anti-BrdU polyclonal	Invitrogen	PA5-32256
Chicken anti-BrdU	Abcam	ab92837
Rat anti-BrdU	Abcam	ab6326
Rat anti-GFAP	Invitrogen	13-0300
Rabbit anti-NeuN	Abcam	ab177487
Rabbit anti-Iba1	Cell Signaling Technology	17198
Rabbit anti-Caspase-3	Proteintech	19677-1-AP
Chicken anti-DCX	Abcam	ab153668
Rabbit anti-SMPD3	EpiGentek	A67638-020
Rabbit anti-SMPD3	Invitrogen	PA5117447
Rat anti-CD31	Invitrogen	MA1-40074
Rabbit anti-α-Tubulin	Proteintech	11224-1-AP
Rabbit anti Ki67 monoclonal	eBioscience	14-5698-82
Rabbit anti-CD63	Invitrogen	PA5-100713
Rabbit anti-TSG101	Invitrogen	PA5-82236
Rat anti-CD68	eBioscience	50-0681-82
Rabbit anti-S100β	Invitrogen	PA5-78161
Rabbit anti-Olig2	Proteintech	13999-1-AP
Rat anti-NG2	Invitrogen	MA524247
Alexa Fluor 488 Goat anti-Rabbit IgG (H+L)	Invitrogen	A-48282
Alexa Fluor 568 Goat anti-Rabbit IgG (H+L)	Invitrogen	A-11036
Alexa Fluor 568 Goat anti-Chicken IgY (H+L)	Invitrogen	A-11041
Cyanine5 Goat anti-rat IgG (H+L)	Invitrogen	A-10525
Goat anti-Rabbit IgG(H+L)-HRP	SouthernBiotech	4050-05
**Virus**		
AAV-CMV-GFP	SignaGen Laboratories	SL100819
AAV5-U6-shRNA(Ctrl)-CMV-GFP	SignaGen Laboratories	SL100822
AAV5-U6-shRNA(Smpd3)-CMV-GFP	SignaGen Laboratories	SL100874

**Chemicals**		
Neurobasal Plus medium	Gibco	A3582901
B-27 plus supplement (50X)	Gibco	A3582801
Epidermal growth factor	MilliporeSigma	01107
Basic fibroblast growth factor (FGF-2)	Sigma-Aldrich	GF003
Heparin	Sigma-Aldrich	H3149-10KU
Gentamycin sulfate	Gibco	613980010
GlutaMAX supplement	Gibco	35050061
Cultrex poly-L-ornithine (PLO) solution	R&D Systems	34-361-0001
Exosome-depleted fetal bovine serum,	Gibco	A2720803
Clarity ECL western blotting substrates	Bio-Rad	1705060S
Paraformaldehyde	Sigma-Aldrich	158127
Normal goat serum	Invitrogen	31873
DAPI	Invitrogen	D1306
5-Bromo-2-deoxyuridine (BrdU)	Sigma-Aldrich	B5002-1G
Ultracentrifugation bottle with cap	Beckman Coulter	355603
Veterinary Gluture topical tissue adhesive	MWI Veterinary Supply	034207
FluoromountG Slide Mounting Medium	SouthernBiotech	010001
GW4869	Selleck Chemical LLC	S76095MG
Dimethyl Sulfoxide (DMSO)	Fisher BioReagents	BP231-100
Leibovitz's L-15 medium	Gibco	11415064
PRONASE protease, Streptomyces griseus	MilliporeSigma	53-702-25KU
Hanks' balanced salt solution (HBSS)	Gibco	J67799AP
Penicillin-Streptomycin	Gibco	15140122
Fetal bovine serum (FBS)	Gibco	A4736301
DMEM/F-12, HEPES	Gibco	11330-032
cis-4-Hydroxy-D-proline (99%)	ACROS Organics	AC204912500
Exosome-depleted FBS	Gibco	A2720803
Opti-MEM reduced serum medium	Gibco	31985-070
Lipofectamin RNAiMAX transfection reagent	Invitrogen	13778-075
TRIzol reagent	Invitrogen	15596026
SYBR Green Supermix	Bio-Rad	1725272
2x Laemmli Sample Buffer	Bio-Rad	1610737
Protease inhibitor cocktail	MP Biomedical	0215883701
2-Methylbutyric acid	Thermo Scientific	AAA11546AC
**Critical commercial assays**		
BCA protein assay kit	Thermo Scientific	23227
SDS-PAGE Gel Preparation Kit	Boster Bio	AR0138
Direct-zol RNA Miniprep Kits	ZYMO Research	R2052

iScript cDNA Synthesis Kit	Bio-Rad	1708891
TriFECTa SMPD3 RNAi Kit	Integrated DNA Technologies	N/A
**Critical tools and consumables**		
Curved #5/45 forceps	Dumont	11251-35
Sterile Cell Strainers	Fisherbrand	22-363-547
24-well confocal plate	Cellvis	P24-1.5P
Transwell membrane insert	Corning	3470
Falcon 35 mm polystyrene cell culture dish	Corning	353001
Micro-osmotic pump	ALZET	1003D
Brain infusion kit 3	ALZET	0008851
**Experimental models**		
Mouse: CD-1	Envigo	Hsd:ICR (CD-1)
**Software**		
ImageJ	NIH	https://imagej.nih.gov/ij/; RRID:SCR_002285
GraphPad Prism 8.4.0	GraphPad	https://www.graphpad.com/; GraphPad Prism, RRID:SCR_002798
NIS-Elements Advanced Research	Nikon	https://www.microscope.healthcare.nikon.com/products/software/nis-elements/nis-elements-advanced-research; RRID:SCR_014329
NanoSight LM10	Malvern Panalytical	https://www.malvernpanalytical.com/en/support/product-support/nanosight-range/nanosight-lm10
200 Series SpectrAA	Agilent Technologies	https://www.agilent.com/en/product/atomic-spectroscopy/atomic-absorption/atomic-absorption-software/spectraa-software
**Others**		
Sliding Microtome	Thermo Scientific	HM 450
The ChemiDoc XRS+ System	Bio-Rad	https://www.bio-rad.com/en-us/sku/1708265-chemidoc-xrs-system-with-image-lab-software?ID=1708265
GTA 120 graphite tube atomizer	Agilent Technologies	200 series; https://www.agilent.com/
Tecnai T20 transmission electron microscope	Thermo Scientific	https://www.thermofisher.com/us/en/home/electron-microscopy.html
Stereotaxic Alignment Instrument	KOPF Instruments	Model 1900
Nikon A1Rsi Confocal System	Nikon	https://www.microscope.healthcare.nikon.com/
Bead mill 4 Mini Homogenizer	Fisherbrand	15-340-164

Table S2. qPCR primers used in this study.

Gene ID	Forward primer sequence	Reverse primer sequence	Source
Cd9	CTCAGTCGGTTGTCGAGTCC	GGCATGGTACAAGCTGGAGT	Integrated DNA Technologies
Cd63	CAGAAGGAGCTGCGGAGAAA	ATCATTCCCACAGCCCACAG	Integrated DNA Technologies
Cd81	GATAGTGACTCTCGCGCCTC	GCTACACCTAGGATCACGCC	Integrated DNA Technologies
Tsg101	ATCCTGGCTGTCCTTACCCA	CCAGGCTTCTTTTGTGCATCTTAT	Integrated DNA Technologies
Ttr	AGCAGGTTTGGAGTCAGCTT	GTTTTAGGAGCAGGGGAGAAA	Integrated DNA Technologies
Smpd3	CCGCGGTCGCTGTAACC	AATCTCTTTGGCCCGAGCTT	Integrated DNA Technologies
Actb	GCAGGAGTACGATGAGTCCG	ACGCAGCTCAGTAACAGTCC	Integrated DNA Technologies
